# Supplementary material for: Underlying structure and measurement invariance by sex of the state trait anxiety inventory: A psychometric analysis in Ecuador
Source: PLoS One. 2026 Mar 27;21(3):e0345773. doi: 10.1371/journal.pone.0345773 (PMC13028510; doi:10.1371/journal.pone.0345773)
Supplement: Attachment — Submitted filename: Response to reviewer 2.odt [file pone.0345773.s004.odt]

Response to reviewers

Additional Editor Comments:

I thank the authors for their reply to my comments. The only outstanding issues that the WLSMV estimator was used to estimate measurement invariance, yet the authors mention inside the manuscript that for scalar invariance the intercepts were found to be equal. However, in ordinal data measurement invariance with WLSMV assumes that intercepts are not defined; instead, item thresholds are estimated in WLSMV measurement invariance. Hence, I invite the authors to explain the measurement invariance steps in greater detail or revise the invariance analyses if thresholds were not held equal and intercepts were estimated.


Response: We sincerely thank you for identifying this crucial methodological detail. You are entirely correct that when analysing ordinal data, intercepts are not defined, and the appropriate parameter to constrain for scalar invariance is the item threshold.

We have revised our measurement invariance analyses to explicitly define all items as ordered-categorical. Consequently, our scalar invariance step now correctly constrains item thresholds rather than intercepts. Furthermore, we estimated the models using theta parameterisation, which permitted the estimation and constraint of residual variances, thereby allowing us to accurately evaluate strict invariance. Finally, latent mean differences were evaluated directly via the Wald test within the strict invariance model. The Analysis plan, Results section, and Table 4 have been updated to reflect these methodological corrections.
